# Camostat Does Not Inhibit the Proteolytic Activity of Neutrophil Serine Proteases

**DOI:** 10.3390/ph15050500

**Published:** 2022-04-20

**Authors:** Akmaral Assylbekova, Anuar Zhanapiya, Renata Grzywa, Marcin Sienczyk, Christian Schönbach, Timo Burster

**Affiliations:** 1 Department of Biology, School of Sciences and Humanities, Nazarbayev University, Kabanbay Batyr Ave. 53, Nur-Sultan 010000, Kazakhstan; akmaral.assylbekova@nu.edu.kz (A.A.); anuar.zhanapiya@nu.edu.kz (A.Z.); christian.schoenbach@nu.edu.kz (C.S.); 2 Division of Medicinal Chemistry and Microbiology, Faculty of Chemistry, Wrocław University of Science and Technology, Wybrzeże Wyspiańskiego 27, 50-370 Wroclaw, Poland; renata.grzywa@pwr.edu.pl (R.G.); marcin.sienczyk@pwr.wroc.pl (M.S.)

**Keywords:** serine proteases, cathepsin G, neutrophil elastase, proteinase 3, camostat, SARS-CoV-2, COVID-19

## Abstract

Coronavirus disease 2019 (COVID-19) can lead to multi-organ failure influenced by comorbidities and age. Binding of the severe acute respiratory syndrome coronavirus 2 spike protein (SARS-CoV-2 S protein) to angiotensin-converting enzyme 2 (ACE2), along with proteolytic digestion of the S protein by furin and transmembrane protease serine subtype 2 (TMPRSS2), provokes internalization of SARS-CoV-2 into the host cell. Productive infection occurs through viral replication in the cytosol and cell-to-cell transmission. The catalytic activity of TMPRSS2 can be blocked by the trypsin-like serine protease inhibitor camostat, which impairs infection by SARS-CoV-2. At the site of infection, immune cells, such as neutrophils, infiltrate and become activated, releasing neutrophil serine proteases (NSPs), including cathepsin G (CatG), neutrophil elastase (NE), and proteinase 3 (PR3), which promote the mounting of a robust immune response. However, NSPs might be involved in infection and the severe outcome of COVID-19 since the uncontrolled proteolytic activity is responsible for many complications, including autoimmunity, chronic inflammatory disorders, cardiovascular diseases, and thrombosis. Here, we demonstrate that camostat does not inhibit the catalytic activity of CatG, NE, and PR3, indicating the need for additional selective serine protease inhibitors to reduce the risk of developing severe COVID-19.

## 1. Introduction

Processing of the severe acute respiratory syndrome coronavirus 2 spike protein (SARS-CoV-2 S protein) is a prerequisite for infection of the target cell and influences the outcome of coronavirus disease 2019 (COVID-19). More precisely, proteolytic cleavage of the S protein by the cell surface serine proteases furin and the transmembrane protease serine subtype 2 (TMPRSS2) is required to fuse the S protein with the target cell membrane. This mechanism is performed in a two-step sequential hydrolysis. The priming cleavage between the S1/S2 interface by furin is followed by an activating cleavage by TMPRSS2 at the S2´ site [1,2,3,4,5]. Hydrolysis of the SARS-CoV-2 S protein by TMPRSS2 remains an essential step in the process of viral entry into the host cell. Indeed, camostat mesylate (camostat), a clinically proven trypsin-like serine protease inhibitor for treating chronic pancreatitis and postoperative reflux esophagitis, inhibits TMPRSS2 and prevents SARS-CoV-2 infection of target cells. As a result, camostat has been repurposed in clinical trials for the potential therapeutic management of COVID-19 [6,7].

In general, the activity of proteases is precisely regulated to avoid harmful degradation of structural or functional proteins by endogenous natural inhibitors, so-called serine protease inhibitors (serpins) [8,9]. The majority of proteases located at the site of infection originate from activated neutrophils. These neutrophils secrete NSPs from granules including cathepsin G (CatG), neutrophil elastase (NE), protease 3 (PR3), and neutrophil serine protease 4 (NSP4) [10]. After an immune response, proteases need to be regulated to a steady-state; however, deregulation of NSPs and their endogenous serine protease inhibitors can result in chronic inflammatory disorders. In particular, the uncontrolled proteolytic activity of, for instance, CatG is involved in autoimmunity, chronic inflammatory diseases, cardiovascular diseases, and thrombosis [11]. Furthermore, it was demonstrated that the abundance of CD15^+^ neutrophils and CatG transcripts was elevated in COVID-19 patients [12], increased concentrations of NE and CatG were determined in nasopharyngeal swabs of patients with SARS-CoV-2 [13], and levels of CatG in plasma were increasingly higher starting with the control group to moderate, severe, and critical COVID-19 conditions [14]. In addition, CatG might also be responsible for priming the S protein at the S1/S2 interface (polybasic sequence) of the proteolytic sensitive activation loop of the SARS-CoV-2 Omicron variant [15]. These observations indicate the relevance of CatG in COVID-19.

Proteases are characterized depending on the catalytic mechanism to hydrolyze the sessile peptide bonds within a protein. Serine proteases, one of the largest groups, encompass the S1 family, which is further divided into three subfamilies: (i) trypsin-like serine proteases with a preference for basic amino acids, for instance, TMPRSS2, (ii) chymotrypsin-like serine proteases which favor bulky hydrophobic amino acids, and (iii) elastase-like serine proteases with small aliphatic amino acids as preferred amino acid residues in P1 which is the position between two amino acids of the sessile peptide bond towards the N terminal end. While CatG possesses both chymotrypsin-like and trypsin-like serine protease capability, NE, as well as PR3, belong to the elastase-like serine proteases [8]. Several inhibitors are available and classified into two major groups (peptide- or protein-based and non-peptide inhibitors) to antagonize the proteolytic activity of serine proteases. Of these, alpha-1 antitrypsin, aprotinin, and chymostatin belong to the first group, and diphenyl esters of phosphonates, β-keto-phosphonic acid, phenol ester, heparin, and boswellic acid derivatives are categorized to the second group [16]. For instance, sunflower-derived serine protease inhibitors (SFTI) and their derivatives are selective CatG inhibitors based on a 14 amino acid cyclic peptide [17,18]. Peptidyl diphenyl phosphonates are an additional class of serine protease inhibitors, including Suc-Val-Pro-Phe^P^(OPh)_2_, which blocks the proteolytic activity of CatG [19]. Furthermore, sivelestat, which was originally postulated as a selective NE inhibitor [20] to treat acute respiratory distress syndrome [21], also inhibits PR3 [22].

Whether camostat inhibits neutrophil serine proteases (NSPs) is not clear. Thus, we investigated the potential inhibition of CatG, NE, and PR3 by camostat.

## 2. Results and Discussion

### 2.1. Activity-Based Protein Profiling of Cathepsin G (CatG) in the Presence of Camostat

**Figure 1:**
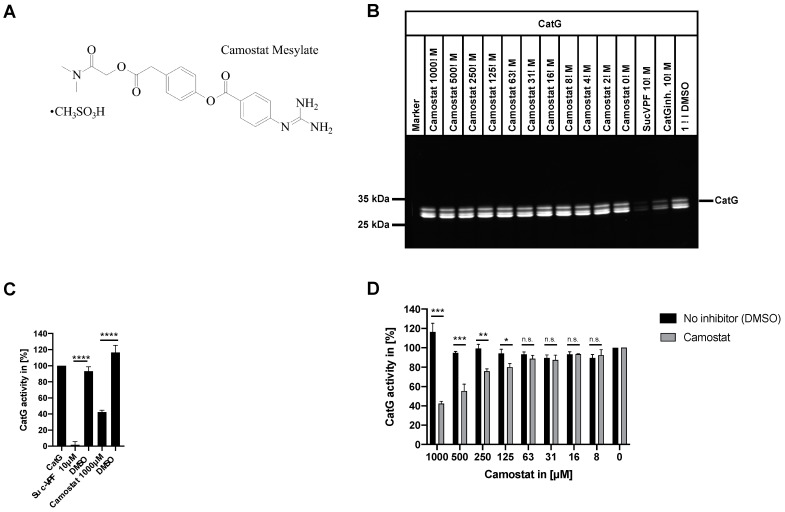
Analysis of the inhibition capacity of camostat towards CatG. (**A**) The chemical structure of the trypsin-like serine protease inhibitor camostat. (**B**) Activity-based protein profiling of CatG by using MARS116-FAM with different concentrations of camostat, Suc-Val-Pro-Phe^P^(OPh)_2_ (SucVPF), or the CatG inhibitor (CatGinh) was performed. DMSO was used as a solvent control. One out of three (*n* = 3) independent experiments is shown. (**C**) CatG activity was analyzed by using the colorimetric substrate 4-(((S)-3-methyl-1-((S)-2-(((S)-1-((4-nitrophenyl)amino)-1-oxo-3-phenyl-propan-2-yl)-carbamoyl)pyrrolidin-1-yl)-1-oxobutan-2-yl)-amino)-4-oxobutanoic acid (Suc-VPF-pNA). In order to validate the assay, CatG, CatG with SucVPF, CatG with camostat, or the vehicle control CatG with DMSO (the volume of DMSO for camostat was higher) were incubated prior to adding Suc-VPF-pNA. The substrate turnover of Suc-VPF-pNA was analyzed at 405 nm. (**D**) CatG was preincubated with different concentrations of camostat or DMSO for 15 min at RT. Afterwards, the colorimetric substrate was added to the assay to lead the enzymatic digest. Three independent experiments are summarized, *n* = 3. *p* < 0.05 (*), *p* < 0.01 (**), *p* < 0.001 (***), *p* < 0.0001 (****), or n.s. = not significant.

Purified CatG was preincubated with different concentrations of camostat and followed by adding the FAM-P1,5D-Suc-Val-Pro-Phe^P^(OPh)_2_ (MARS116-FAM) activity-based probe (ABP), which is a directly 5(6)-carboxyfluorescein (FAM) conjugated peptidyl diphenyl phosphonate, to analyze whether camostat (Figure 1A) inhibits the proteolytic activity of CatG [23]. Since the phosphorus atom of the MARS116-FAM phosphonate warhead is targeted by a nucleophilic attack of the oxygen atom of CatG’s catalytic serine residue, the phosphonate group binds covalently to the serine side chain [24]. Thus, MARS-116-FAM can be used to detect CatG activity in an SDS-PAGE-based assay [24]. As demonstrated in Figure 1B, camostat did not inhibit the catalytic activity of CatG determined by activity-based protein profiling, in contrast to Suc-Val-Pro-Phe^P^(OPh)_2_ (SucVPF) or the CatG inhibitor (CatGinh), which blocked the proteolytic activity of CatG at a concentration of 10 μM.

The turnover of the colorimetric substrate Suc-VPF-pNA by CatG and camostat was used to support the activity-based protein profiling results. CatG was preincubated at different concentrations of camostat, and the hydrolysis of the substrate by CatG was determined. SucVPF or the CatG inhibitor antagonized the proteolytic activity of CatG at 10 μM in contrast to camostat where high concentrations (500 to 1000 μM) were necessary to reduce CatG-mediated hydrolysis substantially (Figure 1C,D). Taken together, these data indicate that camostat does not mitigate CatG activity at reasonable concentrations.

### 2.2. Activity-Based Protein Profiling of NE and PR3 in the Presence of Camostat

**Figure 2:**
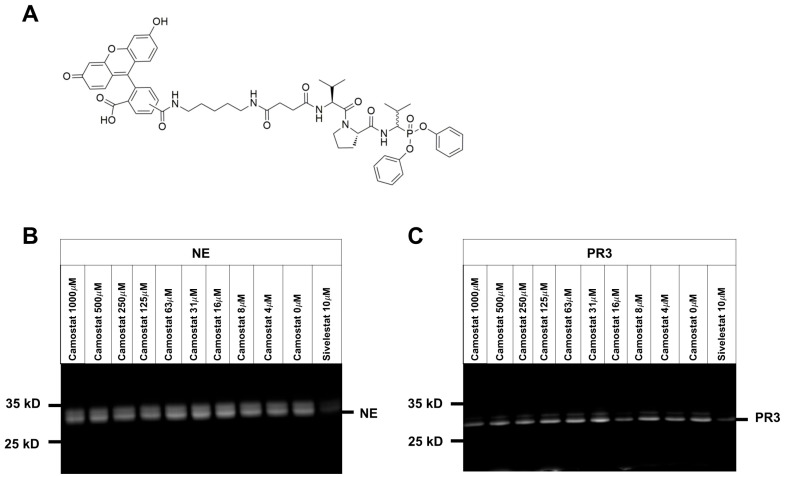
The inhibition capacity of camostat towards NE and PR3 was analyzed by using activity-based protein profiling. (**A**) Structure of activity-based probe FAM-DAPe-Suc-Val-Pro-Val^P^(OPh)_2_ (VPV-FAM). (**B**) Activity-based protein profiling of NE and (**C**) PR3 by using VPV-FAM with indicated concentrations of camostat was performed. 10 μM of sivelestat was used as an inhibitor for NE and PR3. One representative gel out of three (*n* = 3) experiments is shown.

In the next set of experiments, purified NE or PR3 were preincubated with different concentrations of camostat followed by the addition of a FAM-conjugated activity-based probe VPV-FAM. Camostat did not inhibit both NE and PR3 in contrast to sivelestat (Figure 2).

Hoffmann et al. found that 10 μM of camostat reduced infection of primary human lung cells by SARS-CoV-2 [25]. The dosage of 600 mg/day camostat in the SPIKE-1 clinical trial preliminary data resulted in a plasma concentration of 0.2 μM ± 0.1 μM (87.1 ng/mL ± 29.5 ng/mL, https://www.covid-19-Camostat-trial.com, accessed on 17 April 2022). However, in a randomized, double-blinded, placebo-controlled trial with patients receiving 200 mg of camostat three times a day for five days, the clinical outcome of COVID-19 did not improve [26]. Our findings demonstrate that only a very high concentration of 500 to 1000 μM of camostat considerably reduced the turnover rate of the colorimetric substrate but not directly the proteolytic activity of CatG determined by activity-based protein profiling. The dose of camostat, which is used in clinical trials, is also not sufficient to reduce the proteolytic activity of CatG, NE, and PR3 and might partly explain the outcome of the clinical trial for hospitalized COVID-19 patients.

**Figure 3:**
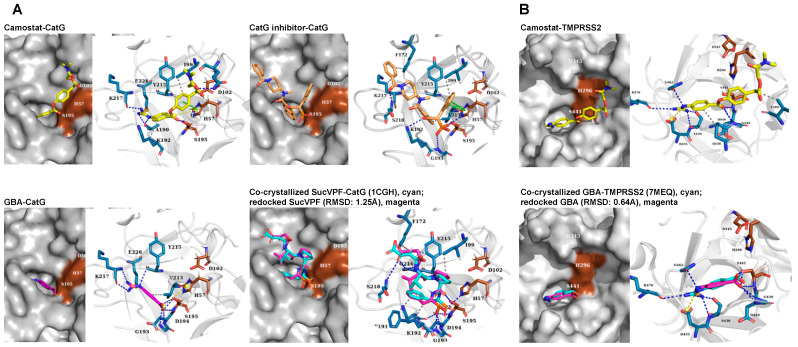
Binding processes of serine protease inhibitors to CatG and TMPRSS2 by using AutoDock4. (**A**) The catalytic center containing the catalytic triad is tinted brown within the surface model of CatG (grey). Interaction of camostat (yellow), GBA (pink), CatG inhibitor (orange), and SucVPF (green) with CatG. Redocking the co-crystalized SucVPF-CatG (1CGH) complex and SucVPF was performed to validate the docking protocol. (**B**) Prediction of TMPRSS2 complexed with camostat. Co-crystallized GBA-TMPRSS2 (7MEQ) and redocking with GBA were attributed for validation. The crystal structures of the complexes were analyzed using the protein-ligand interaction profiler (PLIP). The dashed lines correspond to the molecular interactions formed between the inhibitor and the indicated amino acids of CatG or TMPRSS2. Dashed lines in blue, hydrogen bonds; grey, hydrophobic; yellow, salt bridges; green, pi-stacking interactions.

In order to interpret and correlate the above experimental findings with calculated values of binding free energies and various docking options, a computational method (AutoDock4) was used to predict critical interacting groups between camostat along with NSPs and TMPRSS2. Based on this method, the binding free energy of the inhibitor to the catalytic center of the protease can be calculated [27]. The free binding energy for camostat, the camostat inactive metabolite 4-guanidinobenzoic acid (GBA), and CatG inhibitor in complex to CatG (Protein Data Bank ID, PDB ID: 1CGH) was evaluated. As a result, the molecular docking calculation estimated the following values for camostat-CatG −6.52 kcal/mol, GBA-CatG −4.90 kcal/mol, and CatG inhibitor-CatG −9.65 kcal/mol (Figure 3A and Supplementary Tables S1 and S2). Furthermore, the redocked binding energy of SucVPF to CatG corresponded to −8.75 kcal/mol. Notably, the predicted inhibitor-binding pose of camostat-TMPRSS2 (−8.69 kcal/mol) was supported by GBA-TMPRSS2 (−7.31 kcal/mol) for which X-ray crystallography data are available (7MEQ) [28] (Figure 3B). The camostat’s guanidinium group formed a salt bridge to the TMPRSS2 binding pocket D435. Camostat is further stabilized within the hydrophobic patch forming a hydrophobic interaction with V280. Seven hydrogen bonds are determined in the camostat-TMPRSS2 complex [29].

**Figure 4:**
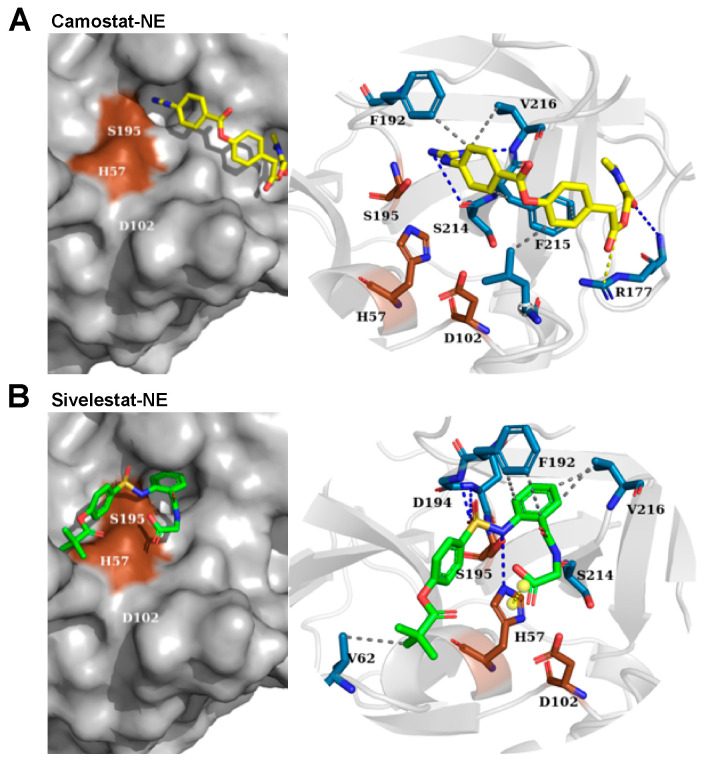
Binding poses of camostat and sivelestat at the catalytic center of NE. Camostat (yellow) and sivelestat (green) were simulated for binding to the catalytic center of NE (1B0F). (**A**) Predicted amino acid residues forming three hydrogen bonds (R177, S214, and V216), three hydrophobic (F192, F215, and V216), and one salt bridge (R177) between camostat and NE. (**B**) sivelestat possesses six hydrogen bonds including H57, G193, D194, S195, and S214, five hydrophobic including V62, F192, and V216, and one salt bridge (H57) with NE. The catalytic triad (brown) within the surface model of NE (grey) and blue dashed lines refer to hydrogen bonds. Hydrophobic interactions and salt bridges are represented by grey- and yellow dashed lines, respectively.

**Figure 5:**
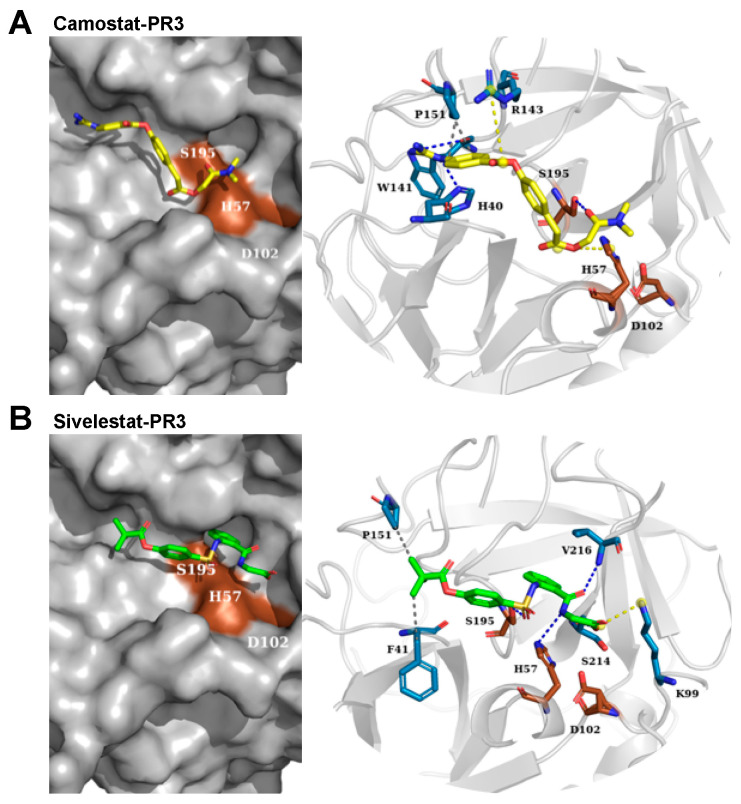
A molecular docking approach was used to determine the binding affinity of camostat and sivelestat to PR3. (**A**) The predicted poses for camostat (yellow) to PR3 (1FUJ) exhibited three hydrogen bonds (H40, W141, and S195), one hydrophobic (P151), and two salt bridges (H57 and R143). (**B**) The ligand-binding affinity prediction of sivelestat (green) results in four hydrogen bonds to PR3 (H57, S195, S214, and V216), two hydrophobic (F41 and P161), and one salt bridge (K99). The catalytic triad (brown), the surface model of NE (grey), blue dashed lines refer to hydrogen bonds, hydrophobic interactions (grey dashed lines), and salt bridges (yellow dashed lines).

Camostat and sivelestat were estimated for their binding free energies to NE and PR3. The binding simulation of camostat-NE was equivalent to −6.35 kcal/mol and camostat-PR3 −6.19 kcal/mol (Figure 4 and Figure 5, and Supplementary Table S3). Sivelestat, which was used as a control inhibitor, exhibited free binding energy to NE of −7.03 kcal/mol and in the case of PR3 −6.50 kcal/mol (Supplementary Table S3). These indicate a lower affinity of camostat to NE and PR3 in contrast to sivelestat. Additionally, camostat topologically points out of the catalytic center of both NE and PR3 (Figure 4A and Figure 5A), which further explains a non-inhibitory capacity of camostat towards NE and PR3.

While each analyzed inhibitor formed hydrogen bonds with the active-site serine residue (S195) of CatG, the number and intermolecular forces of neighboring residues also change the binding energy and influence the inhibition capacity. For example, the hydrogen bonding of SucVPF and CatG inhibitor with H57, K192, and S218 of CatG, absent in both camostat and GBA, might contribute to the lower predicted free binding energy (Figure 3A and Supplementary Table S2). In contrast, the redocking of GBA to TMPRSS2 (7MEQ) and using the docking approach for camostat to TMPRSS2 showed similar hydrogen bonds and salt bridges within the catalytic center (S436, G439, D440, S441, G462, R470, and D435). The estimated free binding energy was −7.31 kcal/mol for GBA-TMPRSS2 and −8.69 kcal/mol for camostat-TMPRSS2, which are comparable values determined for CatG in the presence of CatG inhibitor or SucVPF (Figure 3 and Supplementary Table S2). The inhibition constant (*K*i) was predicted for camostat-TMPRSS2 versus camostat-CatG and was in the order of nanomoles only for camostat-TMPRSS2 (424.27 nM) in contrast to camostat-CatG (16.67 µM) (Supplementary Table S2). In summary, the differences in the estimated free binding energy of the camostat-CatG complex compared to SucVPF and CatGinh explain that camostat does not inhibit the proteolytic activity of CatG in the activity-based protein profiling experiment (Figure 1B). A similar rationale applies to the non-inhibition of NE and PR3 by camostat (camostat-NE, −6.35 kcal/mol and 21.97 µM and camostat-PR3, −6.19 kcal/mol and 29.17 µM, Figure 4 and Figure 5 as well as Supplementary Table S3). In the case of the obtained data from the colorimetric substrate (Suc-VPF-pNA), camostat partly inhibits CatG activity only at high concentrations (500 to 1000 μM), which might be due to the fact that camostat can bind to the catalytic center of CatG (−6.52 kcal/mol, Figure 3A). This is not the case in the activity-based protein profiling, where camostat has to compete with the ABP, MARS116-FAM, which is a component based on the chemical moiety of SucVPF [23] (−8.75 kcal/mol, Supplementary Table S3).

Besides camostat, various other promising compounds have been used in clinical studies to treat COVID-19, including heparin. For instance, Buijsers et al. showed that administering heparin to COVID-19 patients is beneficial. Heparin might reduce endothelial leakage by inhibiting heparinase, which prevents leukocyte infiltration and promotes anti-coagulation via binding to anti-thrombin III [30]. Remarkably, heparin interacts with CatG in an allosteric manner, provoking a non-competitive inhibition of CatG [31]. Interestingly, another study revealed a concentration-dependent effect of heparin on CatG activity that was significantly diminished by 0.29 µM heparin, whereas an increase in heparin concentration slightly reversed this effect [32]. We found that an excess of heparin slightly increased the proteolytic activity of CatG [33]. In particular, further investigations are warranted with the significant number of COVID-19 patients who did not benefit from heparin treatment [34,35].

Alpha-1 antitrypsin blocks the catalytic activity of NSPs and TMPRSS2. The anti-inflammation, anti-coagulation, and serine protease inhibitory characteristics of alpha-1 antitrypsin, evident in the treatment of alpha-1 antitrypsin deficiency, render it an attractive component for the COVID-19 treatment [36,37]. Whether alpha-1 antitrypsin prevents SARS-CoV-2 replication in the target cell has been challenged. Therapeutically acceptable concentrations were only achieved with the serine protease inhibitor aprotinin but not for alpha-1 antitrypsin [38]. Aprotinin was found to effectively restrict the infection of influenza A by disrupting viral hemagglutinin cleavage. Moreover, aprotinin administered in an aerosolized form led to a two-fold reduction of symptoms in patients with seasonal influenza A and parainfluenza [39]. Yet, the commercial therapeutic use of aprotinin was suspended in 2008 due to serious cardiovascular, renal, and cerebrovascular adverse events [40,41]. High levels of NE were found in blood samples of COVID-19 patients, which exacerbates the disease [42]; sivelestat might be a therapeutic option as recommended by [43]. Another encouraging component is boswellic acid, which inhibits CatG and NE [44,45]. Camostat, on the other hand, does not inhibit the proteolytic activity of CatG, NE, and PR3, supporting the need to verify other protease inhibitors to manage COVID-19.

## 3. Materials and Methods

### 3.1. Activity-Based Protein Profiling

Purified CatG from human neutrophils was purchased from BioCentrum, Ltd. (Krakow, Poland). CatG was adjusted to a final concentration of 80 µg/mL and incubated with the activity-based probe FAM-P1,5D-Suc-Val-Pro-Phe^P^(OPh)_2_ (MARS116-FAM, final concentration 20 µM) [23] in DPBS pH 7.4 (no calcium, no magnesium, Gibco by Thermo Fisher Scientific, Karlsruhe, Germany, Cat. No.: 14190-094) for 1 h at RT. Samples, including the inhibitors, were preincubated (15 min at RT) with different concentrations of camostat (camostat mesylate, Cayman Chemical Company, Ann Arbor, MI, USA, cat. no.: 16018, batch 0588008-2), 10 µM of CatG inhibitor (CatGinh, Calbiochem, Merck Chemicals GmbH, Schwalbach, Germany, CAT. NO.: 219372), or 10 µM of Suc-Val-Pro-Phe^P^(OPh)_2_ (SucVPF) [19]. All three inhibitors were dissolved in DMSO (Sigma-Aldrich, Taufkirchen, Germany). Afterwards, the samples were reduced, boiled, and resolved by 12% SDS-PAGE (1 μg of CatG per well). CatG activity was visualized by UV light and the Fusion FX6 Edge software (Fusion FX, Vilber Smart Imaging, Eberhardzell, Germany).

Purified NE adjusted to a final concentration of 40 µg/mL (Cat. No. 16-14-051200, lot no. EH2020-03 from Athens Research and Technology, Athens, GA, USA or cat. no. ab280938; lot no. GR3427395-6 from Abcam, Cambridge, MA, USA) or 40 µg/mL PR3 (cat. no. 16-14-161820-L, lot no. PR32020-01, Athens Research and Technology, Athens, GA, USA) were preincubated with different concentrations of camostat (SigmaAldrich, cat. no. SML0057, batch 0000099204) or 10 µM sivelestat (Tocris Bioscience, Bristol, UK, cat. no.: 3535, batch 3A/247536) at room temperature for 15 min. Afterwards, the activity-based probe FAM-DAPe-Suc-Val-Pro-Val^P^(OPh)_2_ (VPV-FAM, final concentration 10 µM) was added to all samples. The samples were separated by 12% SDS-PAGE and visualized with ChemiDoc Touch Imaging System (Bio-Rad Laboratories, Inc., Hercules, CA, USA).

### 3.2. Turnover of the Colorimetric Substrate

The turnover of the colorimetric substrate 4-(((S)-3-methyl-1-((S)-2-(((S)-1-((4-nitrophenyl)amino)-1-oxo-3-phenyl-propan-2-yl)-carbamoyl)-pyrrolidin-1-yl)-1-oxobutan-2-yl)-amino)-4-oxobutanoic acid (Suc-VPF-pNA) was determined with CatG (BioCentrum Ltd., Krakow, Poland). A total of 4 μg/mL CatG, 4 μg/mL CatG with 10 µM SucVPF, or CatG with camostat. DMSO (Sigma-Aldrich, Taufkirchen, Germany) was used as the vehicle control. The reagents were preincubated in DPBS pH 7.4 (no calcium, no magnesium, Gibco by Thermo Fisher Scientific, Karlsruhe, Germany, cat. no.: 14190-094) for 15 min at RT previous addition of the colorimetric substrate Suc-VPF-pNA (final concentration 200 µM). The samples were measured in a 96-well plate reader (Spectra Max 250, MWG Biotech, Ebersbach, Germany) at 405 nm.

### 3.3. Molecular Docking

CatG (1CGH) and TMPRSS2 (7MEQ), NE (1B0F), and PR3 (1FUJ) were retrieved from the Protein Data Bank (PDB). AutoDock Tools (ADT) was used to remove heteroatoms, add polar hydrogens, compute Kollman and Gasteiger charges, and generate PDBQT formatted files. The ligand structures for camostat, GBA, CatG inhibitor, and sivelestat were obtained from PubChem in SMILES formats. SucVPF was downloaded from the BRENDA database in mol format (Supplementary Table S1). The ligands were prepared using ADT and optimized using the MMFF94 mechanical force field implemented in the Avogadro program and converted into PDBQT format using OpenBabel.

AutoDock4 version (4.2.6) for macOS X (Scripps Research, AutoDock, retrieved from https://autodock.scripps.edu/download-autodock4/, accessed on 3 June 2020) was applied to one ligand (flexible) and one protein (rigid) at a time using the Lamarckian genetic algorithm with extended search parameters GA-run 50 and ga_pop_size 300 limited to 50 poses. The ability of AutoDock4 to reproduce the position of co-crystallized ligands contained in each PDB structure was validated by redocking CatG and SucVPF (1CGH), TMPRSS2 and GBA (7MEQ) (Figure 3, Supplementary data Table S2) as well as NE (Figure 4, Supplementary data Table S3) and PR3 (Figure 5, Supplementary data Table S3). The comparison of the redocked compounds with the co-crystallized ligands showed an RMSD of less than 1.5 Å.

Docking for all proteases was performed at the reported binding sites by adopting a grid size of 40 × 40 × 40 Å along the three axes and a grid point spacing of 0.5 Å. Fifty conformations were generated with AutoDock4. Models with the lowest free binding energy were ranked first upon clustering of similar poses with an RMSD threshold of 2 Å and were used as representative poses shown in the figures. Overall, the best binding conformations for the inhibitors possess the lowest binding free energies and molecular interactions with pivotal amino acid residues. Protein–ligand interactions were visualized and analyzed using PLIP and PyMol. Potential molecular interactions between the compounds and the active-site amino acid residues of proteases, along with the free binding energies and *K*i, are shown in Supplementary Tables S2 and S3. Software and online tools used for molecular docking procedures are listed in Supplementary Table S4.

### 3.4. Statistical Analysis

Bar diagrams, including mean with standard deviation (SD) and *p*-value (<0.01) threshold, were generated with Graph Pad Prism (GraphPad Prism 8 for macOS, Version 8.4.3, 471, GraphPad Software, Inc., San Diego, CA, USA). A one-way ANOVA for multiple comparisons of CatG, DMSO, SucVPF, and camostat (Figure 1C) or unpaired, two-tailed Student’s *t*-test (no inhibitor, DMSO) versus camostat (Figure 1D), was applied.

## 4. Conclusions

**Figure 6:**
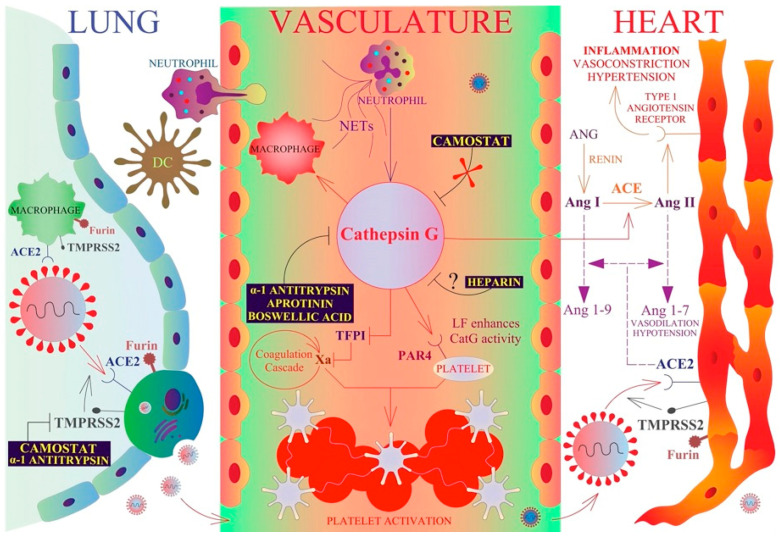
A model of CatG in SARS-CoV-2 infection and inflammation. Activated neutrophils segregate CatG into extracellular space where CatG hydrolyzes the protease-activated receptor 4 (PAR4) on the surface of platelets, which can cause platelet aggregation, coagulation, and thrombosis formation in the vasculature. Thereby, lactoferrin (LF) enhances CatG-mediated platelet activation. Additionally, CatG promotes the recruitment of macrophages and neutrophils to the sites of inflammation and converts angiotensin I (Ang I) to angiotensin II (Ang II) facilitating inflammation, vasoconstriction, and hypertension. Aprotinin, alpha-1 antitrypsin, and boswellic acids are potent inhibitors of CatG, heparin might block CatG activity but in a dose-dependent manner. However, camostat does not inhibit CatG activity (Figure 1). SARS-CoV-2 binds to the ACE2 receptor on type II pneumocytes (lung), alveolar macrophages (lung), and cardiomyocytes (heart), whereas furin and TMPRSS2 prime the SARS-CoV-2-S protein for viral entry into the host cell. Camostat and alpha-1 antitrypsin are potent inhibitors of TMRPSS2 and interfere with productive infection by SARS-CoV-2. ACE2 can convert Ang I and Ang II to Ang 1–9 and Ang 1–7, respectively [11,46].

The proteolytic activity of proteases needs to be tightly regulated to avoid harmful consequences. In such conditions, Figure 6 exemplifies the synergy between CatG and inflammation resulting in cardiovascular diseases, including thrombosis, and the possible therapeutic approach for treating COVID-19 patients.

## Data Availability

Data is contained within the article and supplementary material.

## Supplementary Materials

The following supporting information can be downloaded at: https://www.mdpi.com/article/10.3390/ph15050500/s1, Table S1: Ligand information; Table S2: Amino acids involved in protein–ligand interactions for docking TMPRSS2 and CatG; Table S3: Amino acids involved in protein–ligand interactions for docking of NE and PR; Table S4: Software and online tools used.
