# Supplementary material for: Camostat Does Not Inhibit the Proteolytic Activity of Neutrophil Serine Proteases
Source: Pharmaceuticals (Basel). 2022 Apr 20;15(5):500. doi: 10.3390/ph15050500 (PMC9144258; doi:10.3390/ph15050500)
Supplement: Supplementary file 1 [file pharmaceuticals-15-00500-s001.zip › pharmaceuticals-1650902-supplementary.pdf]

Supplementary data: Table S1. Ligand information

No	Ligand	PubChem CID	Canonical SMILES	Relevance (Inhibition of)
1	**Camostat mesylate (Camostat)** [4-[2-[2-(dimethylamino)-2-oxoethoxy]-2-oxoethyl]phenyl] 4-(diaminomethylideneamino)benzoate;methanesulfonic acid	5284360	N(C)(C)C(=O)COC(=O)CC1C=CC(OC(=O)C2C=CC(NC(=N)N)=CC=2)=CC=1	Clinically proven protease inhibitor for trypsin-like serine proteases
3	**GBA** 4-guanidinobenzoic acid	159772	C1=CC(=CC=C1C(=O)O)N=C(N)N	Inactive metabolite of Camostat Co-crystallized with TMPRSS2, PDB ID, 7MEQ
4	**Cathepsin G Inhibitor I (CatGinh)** [2-[3-[(1-benzoylpiperidin-4-yl)-methylcarbamoyl]naphthalen-2-yl]-1-naphthalen-1-yl-2-oxoethyl]phosphonic acid	9830518	C1C=CC(C(=O)N2CCC(N(C)C(=O)C3C=C4C=CC=CC4=CC=3C(=O)C(P(O)(O)=O)C3C4C=CC=CC=4C=CC=3)CC2)=CC=1	Reversible inhibitor of CatG
5	**Suc-Val-Pro-Phe^{P}-(OPh)$_2$ (SucVPF or 1ZG)**	https://www.brenda-enzymes.org/ligand.php?brenda_ligand_id=18430	C(CCC(O)=O)(=O)N[C@@H](C(C)C)C(N1[C@H](C(=O)NC(CC2C=CC=CC=2)P(OC2C=CC=CC=2)(=O)OC2C=CC=CC=2)CCC1)=O	Irreversible inhibitor of CatG; Co-crystallized with CatG, PDB ID, 1CGH
6	**Sivelestat** 2-[[2-[[4-(2,2-dimethylpropanoyloxy)phenyl]sulfonylamino]benzoyl]amino]acetic acid	107706	CC(C)(C)C(=O)OC1=CC=C(C=C1)S(=O)(=O)NC2=CC=CC=C2C(=O)NCC(=O)O	Reversible inhibitor of neutrophil elastase, also inhibits PR3

Table S2: Amino acids involved in protein-ligand interactions for docking TMPRSS2 and CatG.

**AutoDock Tools**	**Redocked GBA-TMPRSS2 (7MEQ)**<br>**RMSD: 0.64 A**		**Camostat-TMPRSS2**		**Redocked SucVPF-CatG (1CGH)**<br>**RMSD 1.25 A**		**Camostat-CatG**		**GBA-CatG**		**CatG inhibitor-CatG**	
	H-bonds: 9<br>Hydrophobic: -<br>Salt bridges: 1		H-bonds: 7<br>Hydrophobic: 5<br>Salt bridges: 1		H-bonds: 10<br>Hydrophobic: 5<br>Salt bridges: -		H-bonds: 5<br>Hydrophobic: 5<br>Salt bridges: 2<br>Pi-stackings: 1		H-bonds: 8<br>Hydrophobic: 1<br>Salt bridges: 3		H-bonds: 6<br>Hydrophobic: 5<br>Pi-stackings: 1	
**Predicted free binding energy**	-7.31 kcal/mol		-8.69 kcal/mol		-8.75 kcal/mol		-6.52 kcal/mol		-4.90kcal/mol		-9.65 kcal/mol	
**Ki [Temperature = 298.15 K]**	4.41 µM		424.27 nM		385.31 nM		16.67 µM		254.38 µM		83.88 nM	
	**Residues**	**Distance (Å)**	**Residues**	**Distance (Å)**	**Residues**	**Distance (Å)**	**Residues**	**Distance (Å)**	**Residues**	**Distance (Å)**	**Residues**	**Distance (Å)**
Hydrogen bonds	S436<br>G439<br>D440<br>S441<br>G462<br>R470	2.08/3.25<br>2.44<br>2.90<br>1.94/2.63/2.42<br>2.69<br>3.10	S436<br>G439<br>D440<br>S441<br>G462<br>R470	2.16/3.47<br>2.12<br>3.06<br>2.34<br>2.73<br>3.48	H57<br>F191<br>G193<br>D194<br>S195<br>G216<br>S218	2.88<br>2.29<br>1.90<br>3.24<br>**2.94/3.10**<br>2.71/2.08/2.04<br>3.30	Q96<br>A190<br>S195<br>K217	1.70<br>2.10/1.83<br>2.01<br>2.14	G193<br>D194<br>S195<br>Y215<br>K217	3.17<br>3.00<br>2.96/2.29/1.87<br>3.09<br>2.00/3.15	H57<br>K192<br>G193<br>S195<br>S218	2.11<br>2.68<br>3.28<br>**2.20/2.32**<br>1.73
Salt bridges	D435	3.48	D435	3.78	-	-	H57<br>E226	4.16<br>4.29	H57<br>E226	5.49<br>4.30	-	-
Hydrophobic interactions	-	-	Q438<br>V280	3.92<br>3.62	I99<br>F172<br>F191<br>K192<br>Y215	3.59<br>3.94<br>3.44<br>3.13<br>3.63	H57<br>I99<br>K192<br>Y215	3.05<br>3.09/3.66<br>3.76<br>3.44	V213	3.66	I99<br>F172<br>V213<br>Y215<br>K217	3.47<br>3.53<br>3.05<br>3.31<br>3.75
Pi-stacking	-	-	-	-			H57	3.60	-	-	H57	5.19/3.94

Table S3: Amino acids involved in protein-ligand interactions for docking of NE and PR3.

AutoDock Tools	Camostat-NE		Sivelestat-NE		Camostat-PR3		Sivelestat-PR3	
	H-bonds: 5<br>Hydrophobic: 5<br>Salt bridges: 2<br>Pi-stackings: 1		H-bonds: 6<br>Hydrophobic: 5<br>Salt bridges: 1		H-bonds: 3<br>Hydrophobic: 2<br>Salt bridges: 2		H-bonds: 4<br>Hydrophobic: 2<br>Salt bridges: 1	
**Predicted free binding energy**	-6.35 kcal/mol		-7.03 kcal/mol		-6.19 kcal/mol		-6.50 kcal/mol	
***K*i<br>[Temperature = 298.15 K]**	21.97 µM		7.01 µM		29.17 µM		17.26 µM	
	**Residues**	**Distance (Å)**	**Residues**	**Distance (Å)**	**Residues**	**Distance (Å)**	**Residues**	**Distance (Å)**
Hydrogen bonds	R177<br>S214<br>V216	2.8<br>3.43<br>2.48	H57<br>G193<br>D194<br>S195<br>S214	2.70<br>1.91<br>3.58<br>2.83/1.88<br>1.95	H40<br>W141<br>S195	2.25<br>3.40<br>1.79	H57<br>S195<br>S214<br>V216	3.18<br>1,64<br>2.41<br>2.51
Salt bridges	R177	3.74	H57	3.93	H57<br>R143	5.15<br>5.06	K99	3.43
Hydrophobic interactions	F192<br>F215<br>216	3.08<br>3.21/3.22<br>3.86	V62<br>F192<br>V216	3.99<br>3.80/3.84<br>2.23/3.38	P151	3.28/3.44	F41<br>P161	3.84<br>3.63

Table S4: Software and online tools used.

Software (version)/ online tool	Website	Reference
AutoDock 4.2.6	http://autodock.scripps.edu/downloads/autodock-registration/autodock-4-2-download-page/	doi.org/10.1002/jcc.21334
AutoDockTools 1.5.7	http://autodock.scripps.edu/resources/adt	doi.org/10.1002/jcc.21256
Avogadro 1.2.0	http://avogadro.cc/	doi.org/10.1186/1758-2946-4-17
PyMOL 2.4.2	https://pymol.org/2/	The PyMOL Molecular Graphics System, Version 1.2r3pre, Schrödinger, LLC.
PLIP	https://plip-tool.biotec.tu-dresden.de/plip-web/plip/index	doi.org/10.1093/nar/gkab294
PDB	http://www.rcsb.org/pdb/	doi.org/10.1093/nar/28.1.235
PubChem	https://pubchem.ncbi.nlm.nih.gov/	doi.org/10.1093/nar/gkab294
OpenBabel 3.1.1	http://openbabel.org/wiki/Main_Page	doi.org/10.1186/1758-2946-3-33
